# Chromosomal screening of candidate genes associated with F2 chicken birth weight using the random forest algorithm

**DOI:** 10.1016/j.psj.2026.107943

**Published:** 2026-10-11

**Authors:** Donya Khazali, Ali Akbar Masoudi, Rasoul Vaez Torshizi, Ali Maghsoudi, Hossein Bani Saadat

**Affiliations:** Department of Animal Science, Faculty of Agriculture, Tarbiat Modares University, Tehran, Iran

**Keywords:** Machine learning, Genomic evaluation, Body weight, GBLUP

## Abstract

Genomic selection has substantially improved animal breeding by enabling the early identification of genetically superior animals. This advancement is particularly important in poultry breeding, where production efficiency is highly dependent on growth traits such as birth weight. However, the high cost of high-density SNP arrays has stimulated interest in developing cost-effective low-density panels without compromising genomic prediction accuracy. In this study, we employed the Random Forest (RF) algorithm to identify informative SNPs for the development of low-density SNP panels for genomic prediction of birth weight in broilers. Genotypic data from an F2 population derived from reciprocal crosses between Arian broilers and Urmia indigenous chickens were analyzed to identify SNPs associated with birth weight. The objective was to reduce genotyping costs while maintaining prediction accuracy by selecting a subset of highly informative SNPs. After quality control, 45,512 SNPs from 312 F2 chickens were retained for subsequent analyses. SNP importance was quantified using permutation-based variable importance (%IncMSE), and selected marker subsets were evaluated through genomic prediction analyses. The RF algorithm identified informative SNPs with substantial variation in importance among chromosomes. Marker subsets selected using stringent importance thresholds achieved higher genomic prediction accuracy than the complete SNP dataset, indicating that many markers contributed little to prediction performance. Several chromosomes contained few or no highly informative markers, suggesting that they may have limited value for trait-specific low-density SNP panel development. Overall, integrating machine learning-based feature selection with genomic prediction represents a promising approach for developing cost-effective low-density SNP panels to support genomic selection in broiler breeding programs.

## Introduction

Meeting the growing global demand for food under increasingly limited natural resources represents one of the major challenges facing the livestock industry The global population is projected to exceed 9.2 billion by 2050, accompanied by a 35–56% increase in global food demand compared with 2010 levels (van Dijk et al., 2021; Castro et al., 2023). Chickens are among the most efficient sources of animal protein, accounting for more than 30% of global meat production and playing a vital role in food security (FAO, 2022). Consequently, improving poultry productivity through genetic improvement has become a major objective of modern breeding programs. Building on previous studies of Iranian chicken populations (Khazali et al., 2025), understanding the genetic architecture of economically important traits such as birth weight represents an essential next step. Birth weight is a key indicator of subsequent growth performance in chickens. It is closely associated with early survival, post-hatch growth rate, and flock uniformity. Furthermore, optimal hatch weight contributes to improved feed efficiency and growth performance in broilers as well as enhanced laying performance in layer chickens (Willemsen et al., 2008; Van de Ven et al., 2011).

Genomic selection based on dense SNP panels has substantially improved the efficiency of animal breeding programs. However, the high cost of high-density genotyping remains a major limitation, particularly in poultry populations characterized by large breeding populations and short generation intervals. Consequently, the development of cost-effective low-density SNP panels has become an important research priority. The success of genomic selection largely depends on the quality and density of genomic markers. Recent studies have demonstrated that carefully designed low-density SNP panels can provide cost-effective alternatives to high-density arrays (Wolc et al., 2011; Wu et al., 2016). Importantly, many markers included in high-density SNP arrays contribute little to the prediction of economically important traits. (Wolc et al., 2016).

Low-density SNP panels enriched with informative markers linked to quantitative trait loci (QTLs) or causal variants can substantially reduce genotyping costs while maintaining genomic prediction accuracy. Missing genotypes can subsequently be imputed with high accuracy, allowing the reconstruction of genome-wide marker information (Kriaridou et al., 2023). This strategy makes large-scale genotyping economically feasible and may improve genomic prediction by reducing the influence of non-informative markers while retaining the most predictive variants (Tsairidou et al., 2020; Fraslin et al., 2023).

Machine learning approaches have emerged as powerful tools for genomic feature selection and the design of optimized low-density SNP panels. Rather than replacing conventional genomic prediction models, machine learning algorithms can improve their performance by identifying the most informative genetic markers. Algorithms such as Random Forest (RF) can capture complex nonlinear relationships and prioritize SNPs that contribute most to complex traits (Libbrecht and Noble, 2015). This feature-selection strategy facilitates the development of cost-effective SNP panels enriched for predictive markers. When combined with established genomic prediction models such as GBLUP, these refined marker panels may improve prediction accuracy while substantially reducing genotyping costs (Wolc et al., 2016).

A major advantage of machine learning algorithms is their ability to model complex genomic architectures, including weak marker effects, linkage among markers, and non-additive genetic interactions. Furthermore, many machine learning models achieve robust performance with minimal parameter tuning, simplifying their implementation (Montesinos López et al., 2022; Li et al., 2025). These advancements in machine learning methods and genomic selection show promise in improving the precision and effectiveness of breeding initiatives, potentially leading to increased production efficiency and sustainability in poultry breeding programs (Prasad et al., 2024).

Machine learning algorithms, such as Random Forest and other ensemble methods, are revolutionizing the evaluation of genomics in poultry by providing sophisticated tools for detecting significant SNPs associated with growth traits (Montesinos López et al., 2022). These algorithms often offer superior accuracy and robustness compared to traditional linear models, as they can effectively capture non-additive genetic effects and handle large datasets with complex interactions (Ogutu et al., 2012; Libbrecht and Noble, 2015). In the context of chicken breeding, machine learning algorithms demonstrate their utility by identifying the most informative genomic markers. This capability to select key markers directly enhances the precision of genomic predictions, thereby assisting in the selection of superior growth traits and optimizing breeding strategies (Bani Saadat et al., 2024; Ghosh and Cabrera, 2021).

This study aims to utilize the Random Forest (RF) algorithm to identify key SNPs and facilitate the creation of low-density SNP arrays for predicting birth weight in broilers. Distinguishing our approach from conventional whole-genome analyses, we implemented a chromosome-by-chromosome screening strategy. This partitioned analysis significantly reduces computational complexity and eliminates 'genomic noise' from irrelevant regions, which often hampers the sensitivity of machine learning models in large datasets. By isolating each chromosome, the RF algorithm can more precisely pinpoint informative markers while maintaining a uniform distribution across the genome. This methodology mitigates selection bias and ensures that the resulting low-density chips achieve higher predictive accuracy by capturing the true genetic architecture of the trait without the confounding effects of interchromosomal noise.

## Materials and methods

### Data collection

All animal procedures were conducted in accordance with the guidelines for the care and use of animals approved by the Institutional Animal Care and Use Committee of Tarbiat Modares University, Tehran, Iran. The experimental protocol was approved under the ethical approval code IR.MODARES.AEC.1402.072.

### Study population and phenotypic data collection

For this study, an F2 crossbred population derived from the fast-growing Arian line (AA) and the slow-growing Urmia Iranian indigenous chickens (NN) was utilized. The F1 birds were produced by reciprocal crosses AA ♂ × NN ♀ and NN ♂ × AA ♀, and were reared for 12 weeks at the poultry research farm of Tarbiat Modares University, Tehran, Iran. Subsequently, F1 males from each reciprocal cross were mated with 4–8 females from other families to generate the F2 chickens. The F2 generation was individually housed in cages equipped with nipple drinkers and feeders and raised for 12 weeks under identical environmental and nutritional conditions. A total of 312 birds from six different hatches were available. In the present study, birth weight records were analyzed. Birth weight (BW0) of the n = 312 F2 chicks included in the analysis averaged 43.53 ± 3.91 g (mean ± SD), with a coefficient of variation of 8.99% (range 28.97–55.86 g). Prior to the genomic analysis, BW0 was adjusted for the fixed effects of sex, egg weight, and hatch (six hatches), and the adjusted phenotypes were used in the Random Forest and GBLUP analyses. Further details regarding these traits can be found in Emrani et al. (2017).

### Genotyping and genomic data quality control

Blood samples were collected from 312 F2 birds, and genomic DNA was extracted using the salting-out method and stored at −20°C. The quantity and quality of the DNA were assessed using spectrophotometry and agarose gel electrophoresis.

Genotyping was performed using Illumina technology with a commercial 60 K SNP chip in collaboration with Aarhus University, Denmark. To ensure data integrity, the raw dataset underwent quality control (QC) procedures using PLINK 1.9 software (Purcell et al., 2007). Single Nucleotide Polymorphisms (SNPs) were excluded if they exhibited a call rate of < 95%, a minor allele frequency (MAF) of < 5%, or a Hardy-Weinberg equilibrium test p-value of 1 × 10^−6^ (Emrani et al., 2017). After quality control, a total of 45,512 SNPs spanning twenty-eight autosomal chromosomes and 312 birds were retained for the final analysis.

### Random Forest algorithm

Random Forest (RF) is an ensemble learning algorithm that constructs multiple decision trees using bootstrap samples drawn with replacement from the original training dataset, which included genotypic and corrected phenotypic information. For each tree, approximately two-thirds of the observations are used for model training, while the remaining one-third, referred to as out-of-bag (OOB) samples, are reserved for internal validation. At each node of a tree, a random subset of single nucleotide polymorphisms (SNPs) is selected, and the SNP that provides the best split is used to partition the data. Predictions are obtained by averaging the outputs of all trees in the forest (Breiman, 2001). The prediction for a new input (an animal with genotypic information xi but no phenotypic information yi) is given by:

$$f^{B}fr^{\left(X\right)}=\frac{1}{B}\sum_{b=1}^{B}T\left(X,\Psi_{b}\right)$$

where $\Psi_{b}$،​ represents the b-th tree in the Random Forest. Important parameters include the number of variables sampled at each node, the number of trees, and the minimum node size. The importance of each SNP is assessed using permutation methods (Nicodemus et al., 2010; Li et al., 2018). The Random Forest algorithm, implemented in the R package 'Random Forest,' was used to identify significant SNPs on each chromosome.

To optimize the Random Forest (RF) model for genomic data analysis, an out-of-bag (OOB) cross-validation method was employed using the trainControl function from the caret package. The parameter grid for tuning included the number of variables randomly sampled as candidates at each split (mtry), the number of trees to grow (ntree), and the minimum size of terminal nodes (nodesize). The candidate *mtry* values were generally set to 0.5 × (*p*/3), *p*/3, and 2 × (*p*/3), where *p* represents the number of SNP markers on the corresponding chromosome. Candidate values for *ntree* and *nodesize* varied among chromosomes according to the parameter grids provided in Supplementary Table S1. The optimal parameter combination was selected based on model performance.

The selection of informative SNPs (feature selection) was based on the variable importance measure (VIM) generated by the RF model. The importance of each SNP was assessed using a permutation-based variable importance measure (VIM) (Nicodemus et al., 2010; Li et al., 2018). Specifically, the OOB prediction error was first calculated for each tree. Subsequently, the values of each SNP were permuted while keeping all other variables unchanged, and the OOB prediction error was recalculated. The percentage increase in mean squared error (MSE) between the permuted and non-permuted data, averaged across all trees, was used as an indicator of the importance and predictive contribution of each SNP. This analysis was implemented using the randomForest package in R (Breiman, 2001), and significant SNPs were identified separately for each chromosome.

Following permutation-based variable importance analysis within the Random Forest framework, SNPs were ranked according to their associated increase in mean squared error (MSE). The variable importance of each SNP was quantified using the permutation-based percentage increase in mean squared error (%IncMSE) computed from Out-of-Bag (OOB) samples. While uninformative or neutral markers yield near-zero or negative values due to background noise, predictive loci exhibit elevated %IncMSE. Based on the empirical distribution and observed range of importance scores across the dataset, a progressive, data-driven thresholding strategy was implemented (>1%, >2%, and >4%). The mild threshold (>1%) eliminated baseline genomic noise while retaining broad polygenic variation across all 28 autosomes; the moderate threshold (>2%) balanced marker density and removed markers with smaller effects on genomic prediction; and the stringent threshold (>4%) isolated major-effect candidate loci. This objective thresholding ensures that marker retention is governed by true predictive merit rather than arbitrary fixed-number (Top-N) quotas.

Each subset was then independently evaluated in genomic prediction analyses to assess and compare their predictive accuracies. The objective of this comparison was to identify the most effective subset of markers, thereby informing the selection of an optimal marker panel suitable for developing a cost‑effective, low‑density genotyping array. Finally, this genomic prediction accuracy was compared against the baseline accuracy obtained from a genomic evaluation using all SNPs available on the original chip.

### Genomic evaluation

Genomic estimated breeding values (GEBVs) were estimated using genomic best linear unbiased prediction (GBLUP), with the following model (Gianola et al., 2006):

y=μ+g+e

where y is the vector of corrected phenotypes, μ is the population mean, g is a vector of random additive genomic values with g∼N(0, G$\sigma_{g}^{2}$) where G is the additive genomic relationship matrix between genotyped individuals and$\;\sigma_{g}^{2}$ is the additive genomic variance, and e is the vector of random residual effects with e∼N(0, Iσe2), where $\sigma_{e}^{2}$ is the residual variance, and I is the identity matrix. The additive genomic relationship matrix (G) is constructed as $\frac{z\overset{\prime }{z}}{m}$, where Zis the matrix of centered and standardized genotypes for all individuals, and mis the number of markers. Genomic prediction using GBLUP and estimation of genomic heritability were performed for each selected marker subset and the full marker set using the reproducing kernel Hilbert space regression framework implemented in the BGLR package in R (Pérez-Rodríguez and de los Campos, 2022). The Gibbs sampler was run for 50,000 iterations, with the first 10,000 discarded as burn-in. Every fifth subsequent iteration was retained, yielding 8,000 posterior samples for inference.

Genomic evaluation for significant SNPs with positive %IncMSE identified by Random Forest on each chromosome was performed using the 'BGLR' package, and the accuracy of GEBVs was estimated. The evaluation was also conducted for all significant SNPs (merged) and for all SNPs (both significant and non-significant), and the accuracies of GEBVs were compared.

### Genomic evaluation accuracy

Genomic prediction accuracy was evaluated using five-fold cross-validation as follows (Li et al., 2018):

$$\text{Accuracy}=\frac{r_{\text{GEBV}.\text{phen}}}{\sqrt{h^{2}}}$$

Where, $r_{\text{GEBV}.\text{phen}}\;$is the correlation coefficient between the predicted GEBVs of the birds in the test fold and the corrected phenotypes (phen), and $h^{2}$ is the estimated heritability of the trait. The unbiasedness of genomic predictions was assessed using five-fold cross-validation as follows:

$$b_{\text{GEBV},\text{phen}}=r_{\text{GEBV},\text{phen}}\left(\frac{S_{\text{phen}}}{S_{\text{GEBV}}}\right)$$

Where $b_{\text{GEBV},\text{Phen}\;}$ is the regression coefficient of corrected phenotypes on GEBV that shows the unbiasedness of the GEBV, $r_{\text{GEBV},\text{phen}}$ is the correlation coefficient between the predicted GEBVs of the birds in the test fold and the corrected phenotypes, $S_{\text{phen}}$ is the standard deviation of corrected phenotypes, and $S_{\text{GEBV}}$ is the standard deviation of predicted GEBVs.

### Bioinformatics analyses

Following the genomic evaluation analyses for five categories: > 1, > 2, and> 4, all positive SNPs and all SNPs (positive and negative), the genes associated with the SNPs in the category that exhibited the highest accuracy were identified using the Ensembl database. The chicken reference genome assembly bGalGal1.mat.broiler.GRCg7b and Ensembl release 106 were used for gene annotation. Genes located within ±5 kb of the significant SNPs were identified as candidate genes (Cingolani et al., 2012). A total of 260 genes were obtained. The identified candidate genes are presented in Supplementary Table 2.

Furthermore, an analysis of protein‒protein interactions was conducted utilizing the Search Tool for the Retrieval of Interacting Genes/Proteins (STRING) (V12.0) (Szklarczyk et al., 2019) to identify candidate genes. The visualization of the integrated networks was accomplished using Cytoscape software (v3.10.1). The identification of hub genes within the network was carried out based on a degree>5 in Cytoscape. Functional enrichment analysis of the network was conducted using the Gene Ontology (GO) through ShinyGO 0.85.1. The Gene Ontology analysis investigated the biological processes, molecular functions, and cellular components associated with statistically significant genes (FDR < 0.05). ShinyCircos (v2.0) was employed to generate a comprehensive circular visualization, depicting significant body weight-associated genes, their corresponding SNPs, and chromosomal locations. To enhance gene representation capacity, the SNP location Ring (yellow) was duplicated in the circle plot layout.

## Results

### Random Forest algorithm for chromosomal screening

In the RF methodology, the relevance of SNPs transitions from positive to negative values (Yao et al., 2013). The peak positive value in RF denotes a rise in the Percentage Increase in Mean Squared Error (%IncMSE) when the SNP is randomly changed in relation to the prediction error before SNP permutation. The findings from the RF algorithm analysis are illustrated in Table 1, which presents chromosomes, the total of genotyped SNPs, the number of positive, zero, and negative SNPs, as well as the count of SNPs with %IncMSE >1, >2, and >4.

**Table 1:** Chromosome-wise distribution of SNPs according to random forest variable importance. The table shows the number of genotyped SNPs and the numbers of positive, zero, and negative SNPs for each chromosome, together with the numbers of SNPs exceeding the %IncMSE thresholds of >1, >2, and >4. The total row provides the sum across all 28 chromosomes.

CHR	SNPs	Positive	Zero	Negative	>1	>2	>4
1	7441	4070	160	3211	1903	187	7
2	5637	3472	33	2132	1628	294	15
3	4309	2676	28	1605	1309	216	8
4	3508	2121	19	1368	1062	223	11
5	2248	1604	7	637	909	201	9
6	1767	1178	35	554	638	91	1
7	1856	1122	53	681	590	33	2
8	1477	901	65	511	496	55	0
9	1244	1089	0	155	841	471	61
10	1362	1009	2	351	612	208	24
11	1309	1109	0	200	794	385	49
12	1390	939	40	411	522	46	0
13	1206	1018	1	187	737	336	25
14	1062	810	5	247	488	104	3
15	1071	925	0	146	736	423	76
16	18	17	0	1	14	11	3
17	865	602	13	250	334	37	0
18	912	693	7	212	414	77	0
19	859	567	16	276	330	32	0
20	1557	1067	35	455	625	62	0
21	779	664	0	115	485	243	25
22	311	242	0	69	161	64	2
23	618	488	6	124	311	72	1
24	753	621	4	128	431	153	10
25	179	159	0	20	134	92	14
26	669	432	9	228	238	20	0
27	515	377	1	137	216	46	0
28	590	442	4	144	268	67	0
Total	45512	30414	543	14555	17226	4249	346

The examination of the Mean Squared Error of Prediction (%IncMSE) thresholds uncovered distinct patterns regarding chromosomal contributions. At lower stringency thresholds (%IncMSE >1 and %IncMSE >2), all chromosomes were included in the analysis. Conversely, at the more stringent threshold of %IncMSE >4, chromosomes 8, 12, 17, 18, 19, 20, 26, 27, and 28 were omitted due to having zero SNP values, which suggests their negligible contribution to the predictive model at this stricter cutoff. The analysis at the %IncMSE >4 threshold highlighted chromosomes 15, 9, and 11 as the most significant genomic regions, containing 76, 61, and 49 significant SNPs, respectively. These chromosomes contained the largest numbers of RF-selected SNPs at the %IncMSE >4 threshold, suggesting that they may contain genomic regions with relatively high predictive relevance for birth weight in the studied population.

### Genomic evaluation

The genomic evaluation results (Fig. 1) reveal a significant improvement in prediction accuracy as the stringency of SNP selection increases. By narrowing the SNP set from "All SNPs" to those with %IncMSE >4, the prediction accuracy (ACC) surged from 0.46 to 0.81. The genomic prediction accuracy and its associated standard error (SE) were obtained from the five-fold cross-validation procedure. The highest SE for prediction accuracy was observed when all SNPs were included (ACC = 0.4639 ± 0.1407), whereas lower SE values were observed for the prioritized SNP subsets, particularly the >2 (ACC = 0.6685 ± 0.0494) and >4 (ACC = 0.8056 ± 0.0654) categories. Thus, the prioritized SNP subsets showed higher mean prediction accuracy and lower variability across the five cross-validation folds compared with the full SNP set.

**Fig. 1:**
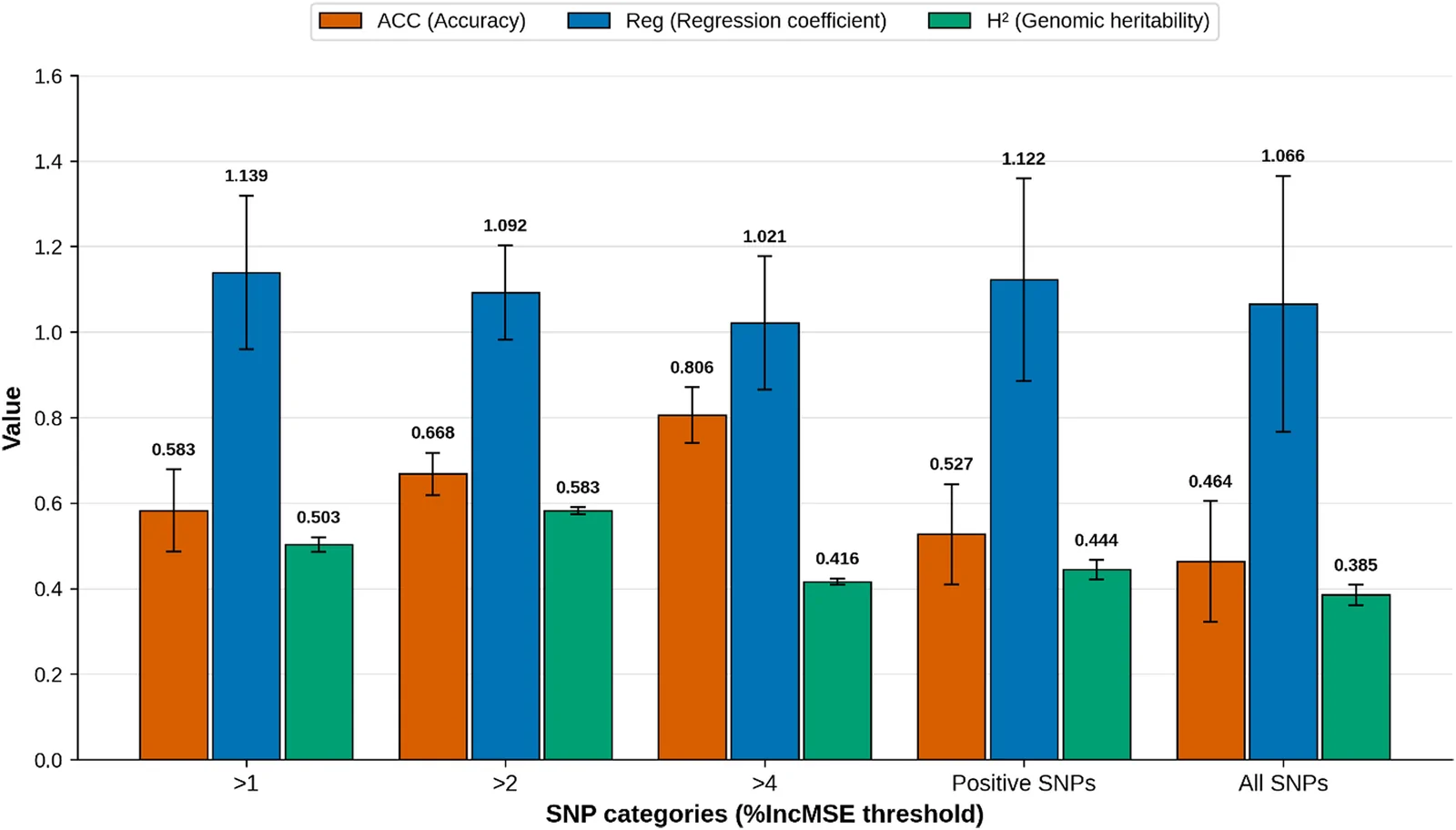
Genomic evaluation of birth weight using SNP subsets selected by the random forest algorithm. Genomic prediction performance was evaluated using the complete SNP set, positive SNPs and SNP subsets defined by %IncMSE thresholds of >1, >2, and >4. Prediction accuracy (ACC), regression coefficient (Reg), and genomic heritability (H²) are presented for each SNP category.

This substantial increase highlights the effectiveness of the Random Forest (RF) algorithm in filtering out "genomic noise"—markers with negligible or confounding effects that often dilute the true genetic signal in whole-genome analyses. The lower accuracy observed in the "All SNPs" category (0.46) can be attributed to the inclusion of thousands of non-informative markers. These markers introduce stochastic noise into the model, leading to over-fitting and reduced generalization power. In contrast, selecting SNPs based on high %IncMSE values ensures that only markers with a robust contribution to the phenotypic variance of birth weight are retained. Furthermore, the heritability (H2) estimates and regression coefficients (Reg) peaked at higher thresholds. Regarding the regression coefficients of phenotypes on genomic estimated breeding values (GEBV), the full marker set (All SNPs) yielded a coefficient of 1.066, accompanied by lower prediction accuracy (0.46) and wider standard error intervals (Fig. 1). Initial marker prioritization slightly shifted the regression slope to 1.122 for Positive SNPs and 1.139 for the %IncMSE >1 threshold, reflecting mild deflation of GEBV variance. However, as the threshold stringency increased, the regression coefficient exhibited a steady and favorable reduction toward the theoretical expectation of unity, declining to 1.092 at %IncMSE >2 and ultimately reaching 1.021 at %IncMSE >4. This near-ideal regression slope (1.02) at %IncMSE >4 coincided with the highest prediction accuracy (0.806), indicating optimal model calibration and negligible bias.

The increase in marker-based heritability from 0.39 (all SNPs) to 0.58 (%IncMSE > 2) reflects the reduction of genomic noise and non-informative loci. Prioritizing informative markers improved the signal-to-noise ratio, allowing the model to capture a higher proportion of the phenotypic variance without the confounding effects of irrelevant loci. The superior performance of the %IncMSE >4 threshold confirms that a carefully curated, low-density SNP panel showed higher prediction accuracy in the present population for specific traits in broilers. This reduction in marker density, while maintaining or even enhancing accuracy, offers a cost-effective and computationally efficient alternative for large-scale genomic selection programs in the poultry industry.

### Bioinformatic analysis

A total of 346 SNPs related to body weight were discovered with a %IncMSE > 4 utilizing the Random Forest machine learning algorithm, which coincided with 260 genes. To enhance comprehension of the functional roles of these genes, gene ontology analysis was conducted. Subsequently, 16 Biological Process and 2 Molecular Function pathways were identified with FDR < 0.05. The majority were primarily linked to body weight (Fig. 2).

**Fig. 2:**
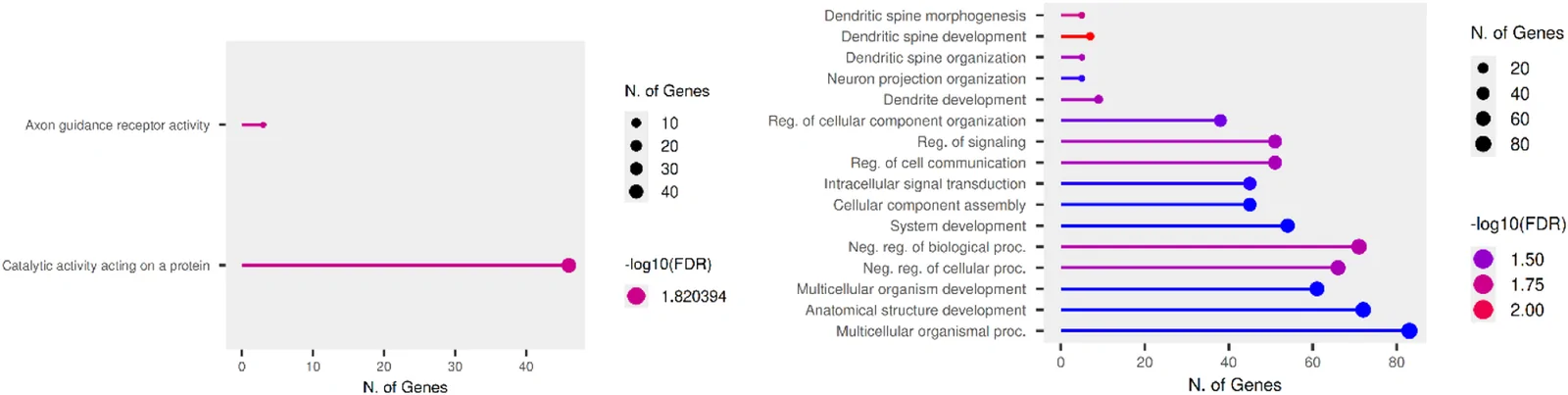
Gene Ontology enrichment analysis of candidate genes associated with birth weight. Gene Ontology enrichment analysis was performed using 260 candidate genes. Significantly enriched GO terms were identified using an FDR threshold of <0.05. The Fig. presents the significant GO terms identified among the candidate genes.

Network analysis of 260 predicted genes was performed using the STRING database and Cytoscape software (v3.10.1). Genes with a degree > 5 were identified as hub genes and are represented by larger yellow-colored nodes in the network. The connections between nodes are indicated by edges, with the color intensity representing the degree of connection: blue (A), pink (B), purple (C), green (D), and yellow (E). The network visualization highlighted several highly interconnected genes, suggesting their potential importance in the biological processes related to body weight regulation in chickens.

The identified hub genes—CDC42, GNB5, PAK2, COPS2, CUL3, EPHB2, EPHB3, TJP1, PIK3CA, and LEO1—occupied central positions within the network architecture. Their extensive connectivity patterns suggest involvement in critical biological pathways governing growth regulation and body weight determination in chickens (Fig. 3).

**Fig. 3:**
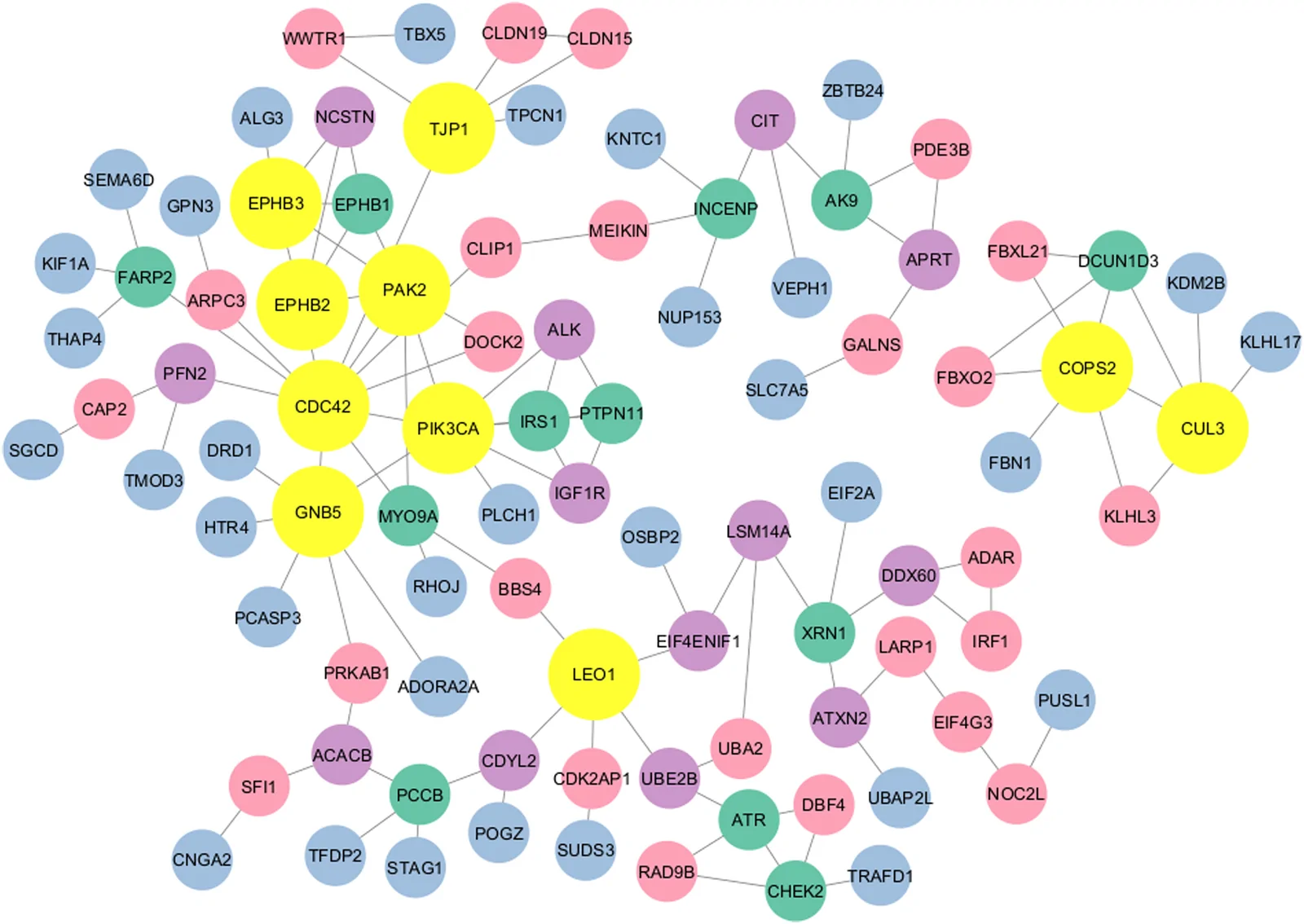
Protein–protein interaction network of candidate genes associated with birth weight. The network was constructed for 260 candidate genes using STRING and visualized in Cytoscape. Nodes represent candidate genes and edges represent interactions between connected nodes, from lowest to highest, in blue (A), pink (B), purple (C), green (D), and yellow (E) colors. Larger yellow nodes indicate hub genes with higher connectivity.

The circle plot provides a comprehensive visualization of the distribution of identified SNPs associated with body weight (Fig. 4). The blue ring represents the number of chromosomes and their respective lengths, while the locations of the identified SNPs related to body weight, indicated by green and pink points, are shown within yellow rings (both yellow rings are identical). These yellow rings are duplicated to enhance the representation of the associated genes. Additionally, the names of the genes linked to these SNPs are displayed around the yellow rings. among the genes present in this plot, some key genes that significantly impact body weight in chickens include GNB5, IGF1R, PAK2, IRS1, EPHB3, COPS2, CUL3, FBN1, LEO1, and EPHB2. These genes are located across various chromosomal regions and play crucial roles in cellular signaling, growth regulation, and metabolic processes.

**Fig. 4:**
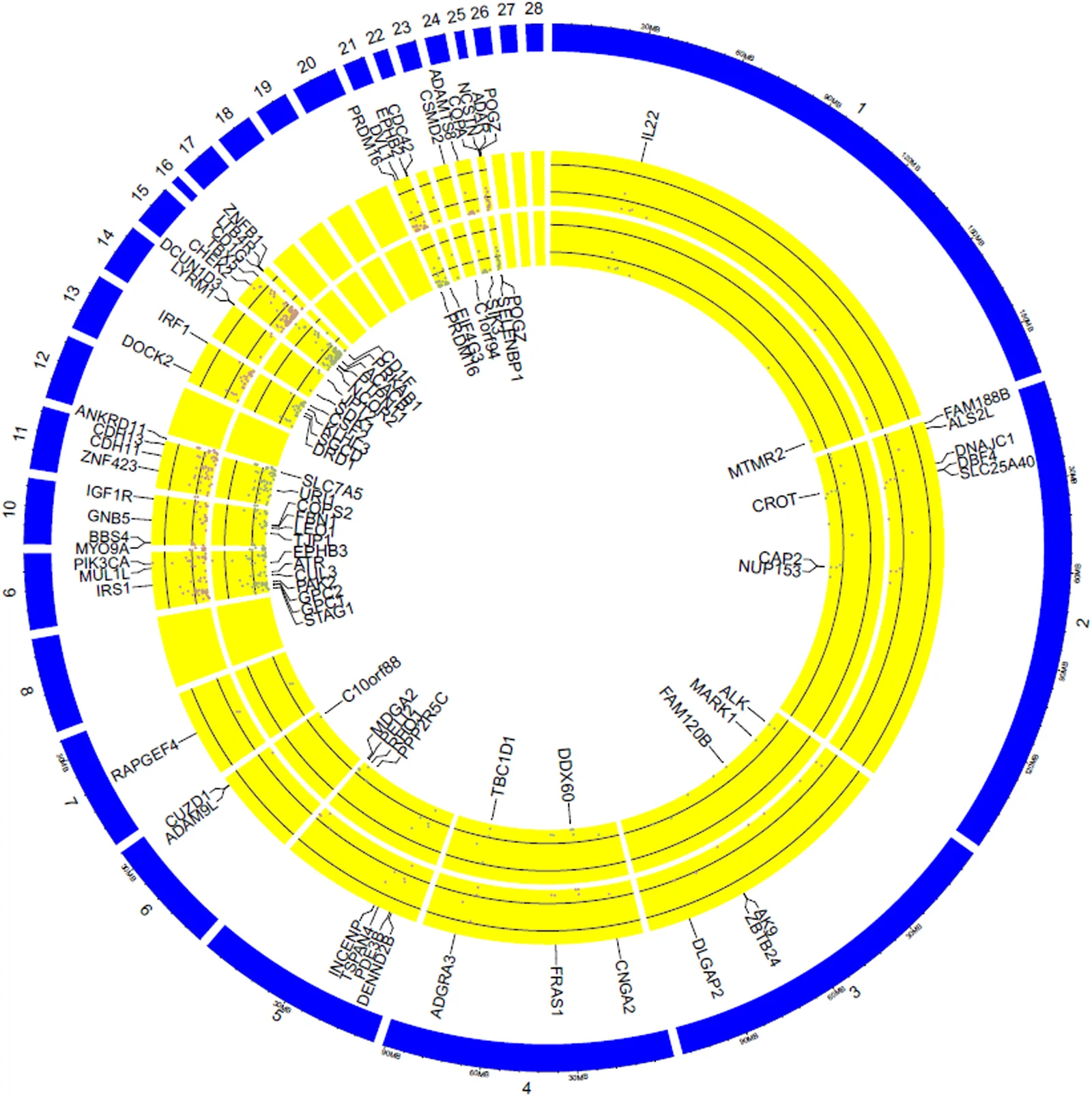
Circos plot showing the chromosomal distribution of selected SNPs and their associated candidate genes for birth weight. The outer blue ring represents the 28 chicken autosomes and their relative chromosome lengths. Green and pink points indicate the genomic positions of the selected SNPs. The yellow tracks indicate the corresponding genomic regions, and the names of associated candidate genes are displayed around the plot.

## Discussion

### Random Forest algorithm for chromosomal screening

The utilization of machine learning within the field of genomics has offered a robust alternative to conventional single-marker association analyses, especially in identifying intricate, non-linear genetic interactions (Libbrecht and Noble, 2015). Our application of the Random Forest (RF) algorithm, enhanced by a stringent permutation-based significance threshold, has effectively progressed from basic SNP discovery to the functional prioritization of genomic regions according to their predictive impact on birth weight. The permutation-based Random Forest analysis uncovered a continuous range of chromosomal contributions to the variation in birth weight, transcending a straightforward binary classification of significant and non-significant chromosomes. This hierarchical perspective better reflects the intricate genetic architecture underlying quantitative traits (Tam et al., 2019).

Although implementing the Random Forest algorithm on a chromosome-by-chromosome basis effectively mitigated the high-dimensionality challenge and prevented large-effect quantitative trait loci on major macrochromosomes from masking subtle polygenic signals on microchromosomes, certain methodological trade-offs warrant consideration. Specifically, partitioning the genomic screening by chromosome restricts the tree-building process from directly evaluating higher-order inter-chromosomal epistatic interactions during the initial feature pre-selection stage. However, given the substantial linkage disequilibrium (LD) within chromosomes in our cross, this partition-based approach prioritized localized non-linear intra-chromosomal dependencies while minimizing genomic noise from unlinked markers. Furthermore, because downstream genomic predictions were implemented using a Bayesian GBLUP framework, the realized genomic relationship matrix derived from the prioritized marker subset effectively captured additive polygenic relationships across the entire genome (Bani Saadat et al., 2024).

The observation regarding the exclusion of nine chromosomes (8, 12, 17-20, 26-28) at the %IncMSE >4 threshold is a crucial discovery. It implies that the genetic influence on birth weight in this cross is not uniformly allocated across the genome, but is instead localized to specific loci.

The identification of chromosomes 15, 9, and 11 as the primary genomic hotspots is noteworthy. At the %IncMSE >4 threshold, chromosomes 15, 9, and 11 contained 76, 61, and 49 RF-selected SNPs, respectively. Comparison with previously reported QTL and GWAS findings provides supportive context for these chromosome-level patterns. Previous studies have reported body weight-related QTL on chromosome 9, including a QTL identified in an F2 intercross between high- and low-growth chicken lines (Nadaf et al., 2009). Similarly, body weight associations have been reported on chromosome 11 in chickens (Jia et al., 2012). For chromosome 15, a recent weighted single-step GWAS identified a 4.63–5.03 Mb region that overlapped QTL for body weight measured at multiple ages in yellow-feather broilers (Fang et al., 2023). Thus, the enrichment of RF-selected SNPs on chromosomes 9, 11, and 15 in the present study is broadly concordant with previous evidence implicating these chromosomes in growth- and body weight-related variation.

The F2 population was derived from Arian broilers and Urmia indigenous chickens, and SNPs were identified according to permutation-based Random Forest importance, whereas previous studies used different populations, marker densities, statistical models, and growth traits or measurement ages. Therefore, the overlap at the chromosome level provides supportive evidence for the potential relevance of these genomic regions in the studied population, but does not establish that the same causal variants underlie the observed signals.

In the present study, SNP importance was evaluated using the permutation-based variable importance measure implemented in the Random Forest algorithm. Rather than defining statistical significance thresholds, which are not inherent to machine learning models, SNPs were evaluated based on their relative importance within the Random Forest framework. By categorizing markers according to increasing %IncMSE values, we assessed the contribution of the most effective SNP subsets—particularly those with %IncMSE > 4—to genomic prediction accuracy (Altmann et al., 2010). This approach surpasses conventional GWAS p-value thresholds in specific situations, as it directly assesses a variable's impact on the performance of the predictive model, thereby capturing epistatic effects that single-locus tests overlook (Brieuc et al., 2018).

### Genomic evaluation

Genomic evaluation results demonstrate that applying a Random Forest (RF)-based SNP pre-selection strategy substantially enhances prediction accuracy for birth weight. We observed a clear threshold-dependent improvement, with accuracy rising from 0.46 (using all SNPs) to 0.81 (retaining only SNPs with %IncMSE >4). This suggests that, for this trait, including the full genome-wide marker set may obscure rather than strengthen the genetic signal. The poor performance of the "All SNPs" scenario likely stems from a large proportion of non-informative markers, which inflate dimensionality and increase noise. In populations with limited sample size, such as the current dataset, such pruning can lead to reduced predictive ability due to overfitting. Similar findings have shown that carefully selected informative SNP subsets can outperform high-density panels in genomic evaluation of growth traits in chickens (Bani Saadat et al., 2024). By prioritizing markers with strong permutation-importance, the RF algorithm effectively captures the underlying genetic architecture while excluding loci with negligible contributions. This targeted selection explains the shift in genomic heritability estimates from 0.39 with all SNPs to 0.58 for the prioritized subset. It is essential to emphasize that the true biological heritability of the population remains unchanged. The higher heritability ​ reflects the elimination of uninformative markers and genomic noise, thereby improving the signal-to-noise ratio in the genomic relationship matrix and allowing loci in high linkage disequilibrium (LD) with causal variants to better capture additive genetic variance. This pattern is consistent with prior poultry studies (Abdollahi-Arpanahi et al., 2014; Bani Saadat et al., 2024), where genomic heritability estimates increased markedly (from 0.30 to 0.59) when using optimized SNP subsets compared to whole-genome dense panels.

From a practical standpoint, the findings demonstrate the potential of constructing low-density SNP panels using RF-derived importance scores for genomic selection in broiler populations. These reduced panels may offer a cost-efficient alternative to high-density genotyping by preserving—and in some cases enhancing—prediction performance. This strategy is particularly valuable for breeding programs with limited financial resources. Nevertheless, the most suitable importance threshold is likely to differ across traits and populations. Therefore, additional studies across multiple generations are needed to evaluate the long-term robustness and consistency of the selected markers.

However, an important limitation of the present study is the population-specific nature of the identified SNPs. The study was conducted in an F2 resource population derived from reciprocal crosses between the Arian broiler line and Urmia indigenous chickens. Therefore, the allele frequencies, linkage disequilibrium structure, and genetic background represented in this population may differ from those of commercial broiler breeding populations. Consequently, the predictive importance of the selected SNPs may not be directly transferable to unrelated commercial lines. Low-density SNP panels developed for genomic selection should therefore be evaluated in the target population, particularly with respect to allele frequencies, linkage disequilibrium, genotyping success, and genomic prediction accuracy (Wolc et al., 2016; Wu et al., 2016). Recent work in chickens has also demonstrated the importance of evaluating low-density SNP arrays across diverse commercial populations (Ma et al., 2026). Accordingly, the SNPs identified in the present study should be considered population-specific candidate markers and a framework for SNP prioritization rather than a finalized universal commercial SNP panel. Validation in independent and commercial broiler populations will be required before practical implementation.

The inverse correlation between SNP number and prediction accuracy was particularly striking. The "All SNPs" approach, representing the conventional genome-wide methodology, yielded the lowest accuracy (ACC = 0.46), likely due to noise from non-informative markers that obscure causal signals (Heinrich et al., 2023). Our findings align with studies in other species, where pre-filtering SNPs using machine learning importance metrics improved Genomic Estimated Breeding Value (GEBV) accuracy by reducing dimensionality and focusing on biologically impactful loci (Song et al., 2025; Lourenço et al., 2024; Wang et al., 2022b). The superior performance of the %IncMSE >4 threshold can be explained by several factors. First, the permutation-based approach identifies SNPs that contribute more strongly to predictive performance, allowing less informative markers to be excluded, effectively filtering out false positives. Furthermore, the near-ideal regression coefficient (Reg = 1.02) for this category substantiates that GEBVs are largely unbiased indicators of true breeding values—a crucial characteristic for effective selection (Legarra et al., 2009).

The regression coefficient of observed phenotypes on GEBVs serves as a benchmark for prediction bias and scale, where a coefficient close to 1.0 indicates unbiased predictions, values >1 reflect inflation (over-dispersion), and values >1 indicate deflation (under-dispersion) (Legarra et al., 2009; Bani Saadat et al., 2024). Although the full SNP set yielded a slope close to unity (1.06), its low accuracy (0.46) reflects the substantial dilution caused by uninformative and. noisy markers. The temporary increase in the regression coefficient at lenient filtering thresholds (reaching 1.139 at %IncMSE >1) can be attributed to initial selection bias and the retention of moderate-effect noisy markers, which mildly compresses GEBV variance. Importantly, progressively filtering out non-essential SNPs restored the regression coefficient to near-perfect calibration (1.02 at %IncMSE >4). This demonstrates that prioritizing highly predictive markers via the Random Forest algorithm helped reduce background noise, avoided over- dispersion, and achieved an optimal balance between maximal predictive accuracy and unbiased GEBV scale (Li et al., 2018).

### Bioinformatic analysis

The Gene Ontology (GO) enrichment analysis indicates that the pathways with the highest significance are not only associated with general metabolic processes, but are notably focused on neurodevelopmental and neuronal morphogenesis pathways. This suggests a previously overlooked connection between nervous system development and the regulation of prenatal growth.

The Biological Process terms that are most significantly enriched pertain specifically to the development of neuronal structures: Dendritic spine morphogenesis, Dendritic spine development, Dendritic spine organization, Neuron projection organization, and Dendrite development. These results are consistent with emerging evidence indicating that neural circuits, especially those involved in hypothalamic development, are vital for regulating body weight and growth. Dendritic spines serve as the primary locations for excitatory synapses within the brain, and their development has a direct influence on the formation and functionality of neural circuits. Recent research has shown that genes responsible for regulating dendritic spine morphogenesis have a significant effect on the development of hypothalamic neurons that govern appetite, energy expenditure, and, as a result, body weight (Grzelka et al., 2023; Xing et al., 2024).

The analysis further revealed multiple significant regulatory pathways, including the Regulation of cellular component organization, Regulation of signaling, Regulation of cell communication, and Intracellular signal transduction. These pathways constitute the essential mechanisms by which genetic information is converted into synchronized growth. The terms "regulation of signaling" and "intracellular signal transduction" are especially pertinent, as they include crucial growth pathways like IGF, WNT, and mTOR signaling, all of which are recognized as vital regulators of body size and tissue development (H’ng et al., 2023; Wang et al., 2022a; Amemiya et al., 2023).

The analysis of Molecular Function identified two distinct activities: Receptor activity and Catalytic activity that act on a protein. The enrichment of receptor activity is especially significant, as receptors play a vital role as gatekeepers for growth factors and hormonal signals. The catalytic activity associated with a protein term probably indicates the significance of post-translational modifications, such as phosphorylation by different kinases, in modulating the function of proteins that participate in growth pathways (Zhang et al., 2025; Shu et al., 2025).

A variety of hub genes were identified within the PPI network, providing additional biological context for the RF-selected genomic regions. Among these, PIK3CA and CDC42 are of particular interest based on their known roles in intracellular signaling and cellular processes related to growth and cytoskeletal organization. PIK3CA encodes the catalytic subunit of phosphoinositide 3-kinase and participates in the PI3K/AKT/mTOR pathway, which regulates cellular growth, proliferation, and metabolic processes (Wiese et al., 2023; Cooley Coleman et al., 2023). CDC42 is a small Rho-family GTPase involved in cytoskeletal organization and intracellular signaling (Zheng et al., 2023). These functional characteristics provide biological context for the prioritization of these genes in the present network analysis.

Nevertheless, the identification of PIK3CA and CDC42 in the present study should not be interpreted as evidence that these genes are causal determinants of birth weight. Their prioritization resulted from the integration of RF-selected SNPs, genomic proximity, and PPI-network connectivity, and no functional validation was performed. Thus, PIK3CA, CDC42, and the other hub genes identified here should be regarded as candidate genes that warrant further investigation rather than confirmed causal genes.

The incorporation of GNB5, a modulator of G-protein-coupled receptor (GPCR) signaling, provides additional biological context for the involvement of extracellular signal transduction in the identified network (Guo et al., 2025; Yang et al., 2016). The identification of EPHB2 and EPHB3 as central hubs is also consistent with their reported roles in cell signaling and neuronal development. These findings provide functional context for the hub genes identified in the PPI network; however, they should not be interpreted as evidence of direct effects on birth weight. Additionally, they are increasingly recognized for their roles in energy homeostasis. Research indicates that EPH signaling plays a crucial role in the development and functionality of hypothalamic neurons responsible for regulating appetite and body weight (Tareq et al., 2025). Additionally, TJP1, commonly known as ZO-1, is a fundamental part of tight junctions that are vital for the establishment of cellular barriers and polarity. Regarding growth, TJP1 can impact the movement of nutrients and the accessibility of hormones through epithelial and endothelial barriers, including the blood-brain barrier, thereby indirectly affecting systemic growth signals (Liu et al., 2020).

The existence of CUL3 and LEO1 highlights the essential function of post-translational and transcriptional regulation. CUL3 serves as a fundamental element of the Cullin-RING ubiquitin ligase (CRL) complex, which is responsible for directing a diverse range of substrates towards degradation, encompassing crucial metabolic and signaling proteins. Consequently, CUL3 is strategically positioned to meticulously adjust the levels of growth regulators (Zhang et al., 2016). LEO1 functions as a subunit of the RNA polymerase II-associated factor (PAF) complex, which plays a crucial role in regulating transcriptional elongation and histone modification. This association links growth regulation to the epigenetic and transcriptional machinery, representing a level of control that is increasingly recognized as significant in complex traits (Laurent et al., 2023). COPS2, which is also referred to as TRIP15, is a part of the COP9 signalosome and plays a similar role in regulating the stability of transcription factors and proteins involved in the cell cycle. (Lykke-Andersen et al., 2003). The integration of these various hubs—encompassing signal transduction, neuronal development, cell adhesion, and protein turnover—into a unified network indicates a strong, multifaceted regulatory framework for birth weight.

In addition to the hub genes identified in our network analysis, a number of other noteworthy candidates arise from the extensive list of 260 genes that exhibit significant potential to affect body weight via various biological mechanisms. Among these PRDM16 serves as a crucial master regulator for the differentiation of brown adipose tissue and thermogenesis. Although avian species do not have the typical brown adipose tissue, it has been established that PRDM16 influences the differentiation of adipocytes and energy expenditure across different species. (Jiang et al., 2022). Current research points to the association of PRDM16 polymorphisms with body composition and growth traits in farm animals, highlighting its potential as a candidate for clarifying genetic variation in body weight through its role in energy metabolism and fat storage (Wang et al., 2012). TBC1D1 acts as a Rab GTPase-activating protein that modulates GLUT4 translocation and glucose absorption in skeletal muscle. This gene has been associated with insulin-mediated glucose metabolism and energy homeostasis (An et al., 2010; Szekeres et al., 2012).

The gene SLC7A5 encodes a major transporter for large neutral amino acids that is essential for nutrient sensing and the activation of mTOR signaling. This transporter plays a pivotal role in the cellular uptake of branched-chain amino acids, particularly leucine, which is a key regulator of protein synthesis and muscle growth (Collao et al., 2022). PDE3B is responsible for the hydrolysis of cAMP and is crucial in the processes of insulin signaling and lipid metabolism. This enzyme holds particular significance in adipose tissue, where it governs both lipolysis and lipid storage (Degerman et al., 2011; DiPilato et al., 2015).

## Conclusion

This research demonstrates the utility of the Random Forest algorithm as an effective feature selection framework to prioritize predictive markers for chicken birth weight. By screening informative genomic regions, this approach provides a cost-effective basis for developing low- density SNP panels without compromising genomic prediction accuracy. Rather than delivering a finalized commercial chip, our findings highlight a prioritized subset of candidate SNPs that significantly refines the input marker density while enhancing predictive performance. Incorporating machine learning into genomic selection frameworks holds promise for mitigating genotyping expenses in large-scale breeding schemes and advancing efficient, sustainable poultry breeding. Future studies should focus on validating these candidate markers across diverse populations and investigating their functional roles to further optimize targeted genomic selection strategies in the poultry industry.

## Author contributions

Donya Khazali contributed to the conceptualization and methodology of the study, data curation, formal analysis, software implementation, visualization, and drafting of the original manuscript. Ali Akbar Masoudi contributed to the conceptualization and methodology of the study, supervision, validation, funding acquisition, and critical revision of the manuscript for important intellectual content. Rasoul Vaez Torshizi contributed to the provision of resources, investigation, supervision, and critical revision of the manuscript. Ali Maghsoudi contributed to the investigation, methodology, and critical revision of the manuscript. Hossein Bani Saadat contributed to the methodology, software implementation, formal analysis, data curation, validation, and critical revision of the manuscript for important intellectual content. All authors reviewed and approved the final version of the manuscript and agree to be personally and publicly accountable for their contributions to the work, including ensuring that questions related to the accuracy or integrity of any part of the work are appropriately investigated and resolved.

## Disclosures

The authors whose names are listed immediately below declare they have no competing interests and if any, the authors confirm that have stated this conflict of interest in the footnotes, Conflict of Interest or Acknowledgments section of manuscript. In addition to, this manuscript has not been submitted to, nor is under review at, another journal or other publishing venue.

## Footnotes

Supplementary material associated with this article can be found, in the online version, at doi:10.1016/j.psj.2026.107943.
